# Reform in the Lunacy Laws

**Published:** 1886-02

**Authors:** James B. Silkman


### REFORM OF THE LUNACY LAWS.

BY JAMES B. SILKMAN.

No. 2.

It is charged that some of the statements in my first brief, prefatory article in regard to the rapid increase of insanity, both in this state and in our whole country, are alarming if not questionable. Let us see if the figures of reliable statistics do not more than bear me out? By the United States census of 1860 our population is set down at a trifle under 31½ millions, while that of 1870 is made a trifle over 38½ millions, showing an increase in that decade of 22 3- 5 per cent.; but the increase in the number of the insane during the same period was from 24,000 to 37,400, more than 55 per cent., or about 2½ times the ratio of increase of population; and instead of one insane person to every 1,300 in 1860, it becomes one to 1,030 in 1870. Again, our population by the census of 1880 is placed at 50,155,700, showing an increase for the last decade of 30 per cent, while the increase in the number of the insane for the same period has gone up from 37,400 to 92,000, or 145 per cent., almost quintupling the ratio of increase of population; and, instead of one insane person in 1,030 in 1870, it becomes in 1880 one in 545.

And whereas I had only roughly estimated the ratio of the increase of the insane as quadrupling that of population for the last 15 years, I feel safe in the conclusion from the above and, other statistics at hand, that, if a census were now taken my statement would be far below the real figures.

Let us now turn to the Federal census of New York State alone. In 1870 the population was 4,383,000 (nearly) and the total number of insane 6,353, or one to 689; by the census of 1880 the population was nearly 5,083,000, and the number of insane 14,111, or one to 360. Here, indeed, we see an alarming rate of increase, for while the increase of population for the decade shows a trifle under 16 per cent., that of the insane exhibits an increase of over 122 per cent., and prompts the inquiry whether many public institutions of this class do not tend to cultivate insanity rather than to cure it.

If we still further narrow the limits of our observation to one only of the many fields covered by the State Board of Charities, whose functions do not reach beyond public and private asylums, and examine the reports made up under the supervision of that accomplished gentleman and indefatigable worker, Hon. Chas. S. Hoyt, we find by the report of last month made up to Oct. 1, 1885, that there were 12,707* insane under the care of this Board, an increase of 584 during the current year; and by examining the reports from 1880 onward we find the following annual increase, Oct. 14, 1880, over the previous report 522; for 1881, 520; for 1882, 648; for 1883, 638; and for 1884, 780. If again we compare the total number in those institutions as reported to Dec. 31, 1874, 6,279, with the last report 12,707, we find an increase in ten years and ten months of over 102 per cent.; while the census of the entire population as above set forth shows only 16 per cent. increase from 1870 to 1880; and for the same period of ten years and ten months it certainly could not exceed 20 per cent. Here again we see the ratio of increase of insanity has more than quintupled that of population; another frightful exhibit for the contemplation of the taxpayer, as well as for the philanthropist and legislator; for in this last catagory of figures, the patients are all supported, in whole or in part, at the expense of the State.

The question obviously arises in the reader's mind? How are we to learn the number of insane in private families? Except when the census taker furnishes it we can only approximate the aggregate by a random estimate. By the Report of the State Board of Charities, it was estimated that in 1884 there were 3,000 under family care, or a total for that year of 15,000. By the Report to our Legislature, January 1st. 1885 by Hon. Stephen Smith, M. D., State Commisioner in Lunacy, our most reliable authority on these subject, it appears, as stated in *Medico Legal Journal*, by its Editor Clark Bell Esq., that there were between 4.000 and 5.000 then in family care with a total of between 16.000 and 17.000 insane.

And by a careful comparison of the Reports from both of the above named official sources for the period of 10 years and 10 months ending Oct. 1st 1885. I find that the average annual increase of the insane under their care, *i. e.* in asylums weas a fraction over 593. Even the census taker is not supposed to make a complete report, for relatives will not generally be found over-zealous to help swell the aggregate number, and hence allowance must be made by adding a considerable percentage to the returns found in the census tables. If the ratio of increase outside of asylums is equal to that shown above, which (for reasons hereafter to be given and already suggested,) I do not believe, the foreshadowing, total increase would be astounding. On the basis of the State Board's, Reports for the last two years the grand total in 1893 will be considerably above 25,000. Again the questions naturally arise: What preparation shall we make for such results? What shall we do? These will be answered in a future number of this journal. Another question naturally arises, if these things continue indefinitely, where shall we find a sufficient number of attendants and physicians to care for the insane? Indeed some writer has raised the question whether all persons have not a spark of insanity in them. In my remarks before the Committee of our State Legislature I said the whole human family may be supposed to stand in one line, and that from an unknown point in which all on the left were *insane*, with a streak of sanity, and all on the right *sane* with a streak of insanity, but that point was moving very rapidly to the right, and at the rate which it is now moving the time must ultimately arrive when we shall be compelled to resort to the better regulated States of Massachusetts and Pennsylvania for a sufficient number of sane atendants to take care of the Lunatics in the Empire state.

It may seem quite paradoxical that one who is neither an alienist or a physician should presume to enlighten the readers of this journal in regard to the manangement of the insane; it is not the management, but the mis-management—the neglect of proper supervision and *cruel* "treatment" that I am to speak of. For every wrong there is supposed to be in law, and there should be a feasible and proper remedy: and to point out the former and suggest the latter is the function of the lawyer as well as that of the alienist. There are different points of view to most subjects—different sides to most questions; to this there is an outside and an inside view; and it must be borne in mind that most of those who have written on Lunacy Reform have been superintendents or alienists writing from their one point of view, and that a very narrow one. In one sense they have written from the outside, while I purpose to write from the inside, what I heard with *my* ears—saw with *my* eyes--suffered in my own soul, *pars quorum fui*. The fable of the Lion and the artist seems applicable here. When the Lion saw that the painter had placed the man on top in the contest, he observed that, if he had made the picture, the condition of things would have been reversed. I am not disposed to lionize myself, but I am morally certain to paint things very differently from what is usually gotten up by many superintendents to mis-lead legislative bodies and state officials. As heretofore intimated, by far the greater number of wrongs done and crimes committed in asylums come under the head of supervision and management, and even here the difficulty rests not entirely on the inadequacy of the present laws, but is due in a large measure to the neglect, on the part of supervising managers and State officials to enforce existing laws. Many lesser abuses have been corrected by them, but I have no recollection of a single case where the release of a sane person has been effected by any such official. And it is a matter of regret that more ample authority is not given to our Excellent State Commissioner in

Lunacy, Hon. Stephen Smith, in this regard.

At the opening of the Session of the Legislative Assembly of this State for 1883, I caused a resolution to be adopted "that the Com- "mittee on State Charitable Institutions be instructed to enquire into "and investigate the entire management of the State Asylums for the insane," etc., etc. But as no special attorney was appointed, the investigation was confined almost entirely to the Utica Asylum with which I was somewhat familiar. The result of this investigation was a report comprising about 600 pages, which was ordered to be printed; the evidence however, was so much more damaging to that Asylum than anything previously given that its publication was, by some means suppressed. But the murder of the patient, Hughes, by the attendants in that Asylum, which occurred early in the session of the legislature of 1884, called for a special committee of investigation and aroused their curiosity as to the disposition of the Report of 1883; it was hunted up, again ordered to be printed, and placed in the hands of the State Printer. Again means were found by interested parties to suppress this report, and it so remains to this day. Perhaps no better or stronger proof can be adduced or called for, of the importance to Utica Asylum to have this report concealed from the public, then the statement forced from the *Utica Herald*, well-known as the organ and mouth-piece of Dr. John P. Gray and the Utica Asylum, that "*the testimony was very damaging to Utica Asylum.*"

Very many know by sad and terrible experience that having once entered the walls of an Asylum the patient has small chance of again entering the world. He is more positively a prisoner than a felon in our State prisons; for here his enemies have no further power over him; and, as each day brings him 24 hours nearer the outer door, his spirits are sustained by the certainty of coming relief. Indeed, the State prisoner obtains a far wider and deeper sympathy from the public than the unfortunate insane. The former is, by custom presumed to be subjected not only to the most rigorous infliction of the penal laws, but to all the incidents of their abuse; while the alleged insane are supposed to be in a charitable institution, under the kindly care of warm-hearted philanthropists—an institution conducted on moral principles and with the Christian banner hanging from its outer walls. The former, in committing a felony, virtually contracts with the State authorities, that, if arrested and convicted —if he can neither escape nor secure a pardon, he will take the chances, not only of the legal penalty due to his crime, but also of all its concomitants of cruelty and brutality which invincible custom has attached thereto. But the insane and the alleged insane are supposed to be incapable of making any contract whatever. They are merely wards of the State, and whether they suffer abuse, cruelty, brutality or death, their lips are alike sealed. The patient cannot write to any person except the "friend" or "relative" who brought or sent him to the asylum, or to those to whom his "friend" or "relative" has given permission to write; nor can the patient be seen, unless the visitor comes fortified with an order of the Supreme Court, or a request in writing from the "friend" or "relative" to this effect. An insane asylum is not intended for, nor should it be longer permitted to be used as a penal institution or an "Inebriate Asylum" where patients are sent as a punishment for crime or the reformation of habits. The pertinency of these simple almost axiomatic truths have been fully illustrated in a series of letters to the Syracuse *Herald* by the writer of this article, giving his experiences in Utica Asylum; nor has any atempt been made to controvert a single one of the long series of indictments against that institution, covering a dozen columns of that paper. I speak of Utica Asylum in particular, because I know more about it, having made it a special study; as a chemist would take a bottle of water from a standing pool or a mineralogist a small piece of ore for analysis and thus discover the elements of the entire mass; and for another reason do I specially allude to Utica; on account of its high standing for a long period, both at home and abroad; if its management fails to meet the requirements of this age, the argument *a fortiori* will readily be supposed to apply to many other institutions of more doubtful reputation; but in fact, with a few notable exceptions here and there, they are generally supposed to hang together like a bundle of chestnut burs. In a future number I will state my list of abuses; some proofs of their existence, and subsequently the needed remedies; and conclude this article with a quotation from the unanimous Report of the special committee on Utica Asylum made to the Legislature of this State in 1884, of which Hon. Walter Howe was chairman; a committee composed of members of every shade of party politics. "The entire unanimity with which all the supervisors and attendants now connected with the institution (Utica Asylum), some of whom have been there many years *deny the fact of such assaults*, (by attendants upon patients) is to the committee a subject of suspicion, and when read in connection with the testimony of the others (many patients) before referred to, indicates to the committee *such a reckless disregard of truth* in this respect as to greatly impair their credibility upon other subjects. It is therefore, the *unanimous belief of the Committee that the attendants do from time to time, treat the patients with reckless and wanton roughness, and at times with a cruelty that is simply outrageous considering the helpless mental condition of the patients.*"

*To be Continued.*

* The Report of the State Commissioner in Lunacy, to January, 1886, gives the total number under his care 12,848, and an increase for last year 617.

